# Software tool for internal standard based normalization of lipids, and effect of data-processing strategies on resulting values

**DOI:** 10.1186/s12859-019-2803-8

**Published:** 2019-04-29

**Authors:** Jeremy P. Koelmel, Jason A. Cochran, Candice Z. Ulmer, Allison J. Levy, Rainey E. Patterson, Berkley C. Olsen, Richard A. Yost, John A. Bowden, Timothy J. Garrett

**Affiliations:** 1 Department of Chemistry, University of Florida, 214 Leigh Hall, Gainesville, FL 32611 USA; 2 College of Engineering, University of Florida, 412 Newell Dr., Gainesville, FL 32611 USA; 3 Hollings Marine Laboratory, National Institute of Standards and Technology, 331 Ft. Johnson Road, Charleston, SC 29412 USA; 4 Clinical and Translational Science Institute, University of Florida, 2004 Mowry Road, Gainesville, FL 32610 USA; 5 College of Public Health & Health Professions, University of Florida, 1225 Center Dr., Gainesville, FL 32611 USA; 6 Department of Pathology, Immunology, and Laboratory Medicine, College of Medicine, University of Florida, 1395 Center Dr., P.O. Box 100275, Gainesville, FL 32610-0275 USA; 7 Center for Environmental and Human Toxicology, Department of Physiological Sciences, College of Veterinary Medicine, University of Florida, Gainesville, FL 32601 USA

**Keywords:** Lipidomics, Data-independent analysis, Mass spectrometry, High resolution mass spectrometry, Liquid chromatography, Lipid quantification, Relative quantification, SRM 1950, Peak picking, MZmine

## Abstract

**Background:**

Lipidomics, the comprehensive measurement of lipids within a biological system or substrate, is an emerging field with significant potential for improving clinical diagnosis and our understanding of health and disease. While lipids diverse biological roles contribute to their clinical utility, the diversity of lipid structure and concentrations prove to make lipidomics analytically challenging. Without internal standards to match each lipid species, researchers often apply individual internal standards to a broad range of related lipids. To aid in standardizing and automating this relative quantitation process, we developed LipidMatch Normalizer (LMN) http://secim.ufl.edu/secim-tools/ which can be used in most open source lipidomics workflows.

**Results:**

LMN uses a ranking system (1–3) to assign lipid standards to target analytes. A ranking of 1 signifies that both the lipid class and adduct of the internal standard and target analyte match, while a ranking of 3 signifies that neither the adduct or class match. If multiple internal standards are provided for a lipid class, standards with the closest retention time to the target analyte will be chosen. The user can also signify which lipid classes an internal standard represents, for example indicating that ether-linked phosphatidylcholine can be semi-quantified using phosphatidylcholine. LMN is designed to work with any lipid identification software and feature finding software, and in this study is used to quantify lipids in NIST SRM 1950 human plasma annotated using LipidMatch and MZmine.

**Conclusions:**

LMN can be integrated into an open source workflow which completes all data processing steps including feature finding, annotation, and quantification for LC-MS/MS studies. Using LMN we determined that in certain cases the use of peak height versus peak area, certain adducts, and negative versus positive polarity data can have major effects on the final concentration obtained.

**Electronic supplementary material:**

The online version of this article (10.1186/s12859-019-2803-8) contains supplementary material, which is available to authorized users.

## Background

Lipids partake in diverse and critical biological roles, such as in cell signaling [1–3], membrane function and integrity [4], alveoli functioning [5], energy storage [6], and water retention in the skin [7] and eyes [8]. These varied biological roles are achieved through the vast heterogeneity and complexity in lipid structure, distribution, and concentration. For example, individual lipids can differ by over six orders of magnitude in concentration [9], while chemical and physical properties can vary in polarity, structural orientation, and charge state (e.g., charged, zwitterionic, and neutral lipid species). Advancements in mass spectrometry and the advent of electrospray ionization (ESI) have enabled researchers to begin to detect this wide diversity of lipids; however, quantification of these detected lipids is challenging due to their dynamic range and breadth of chemical properties.

For quantitation in lipidomics, either relative, semi-quantitative or absolute/accurate quantification can be performed. Absolute/accurate quantification typically employs matrix-matched external calibration curves and/or isotopically labeled internal standards for each lipid quantified. This quantitative approach has limited application to untargeted lipidomics analyses due to the enormous diversity of the lipidome, limited availability of appropriate standards to cover this diversity, and the cost associated with purchasing hundreds of standards [10–12]. Semi-quantification is used when stoichiometric differences between lipid species is of interest, but exact quantitative levels within 10–20% are not obtained. Often both an internal calibrant and external calibration are used for semi-quantification [10, 13]. Relative quantification is often sufficient where relative changes are of concern, for example between diseased and control populations [14], but stoichiometric differences between lipids are not needed. Relative quantification, which does not employ a calibration curve, and involves the addition of a smaller set of internal standards representative of the classes of lipids analyzed, is the most commonly used approach for quantitation in untargeted lipidomics experiments.

Currently limited standards exist for quantification; deuterated standards (often deuterated at the terminal carbons of fatty acyl chains for easily predicted fragment mass shifts) and odd chain standards or other standards with fatty acyl chains which do not exist in the study system can be used. The selection of the most appropriate internal standard to best represent a lipid feature can be challenging. The dynamic range and ionization efficiency are both important for quantitation, and can differ depending on the lipid molecule’s structure, more specifically lipid class, degrees of unsaturation, and number of carbons in fatty acyl chains. Lipid class generally has the greatest effect on ionization efficiency. Previous reports have shown that lipid internal standards spiked into samples at the same concentration have orders of magnitude differences in intensities across different classes [15]. Therefore, lipids should generally be quantified using standards from the same lipid class. To account for the number of carbons and degrees of unsaturation in fatty acyl chains, which both lead to an increase in ionization efficiency [15], two or more lipid standards per class, each with different carbons and degrees of unsaturation is suggested for polar lipids [16]. For neutral lipids, where fatty acids play a greater role in ionization efficiencies, response curves based on a wide range of internal standards is often employed [11, 16]. The differences in carbons are often a more significant contributor to ionization efficiency than that of unsaturation at low concentrations, while at high lipid concentrations the effect of unsaturation on ionization efficiency becomes more pronounced [15].

In addition to lipid structure and sample composition, overlapping chromatograms, ion suppression, large dynamic ranges in lipid concentration, extraction procedure [16], and other methodological and instrumental factors can affect the amount of lipid signal observed [10]. Ultra-high performance liquid chromatography (UHPLC) and high-resolution mass spectrometry (HRMS) can be employed to increase specificity. HRMS reduces the overlap of mass spectral peaks from isobars, resulting in a decrease in residual standard deviations of measurements and more accurate peak integrations, which are used for more accurate quantification [17]. Chromatography also reduces the possibility of peak overlap by adding an orthogonal dimension of separation, and can reduce ion suppression by separating lipid classes and species, reducing the probability of high abundant lipid classes suppressing low abundant lipid classes [16].

Problematic issues arise in reverse phase (RP) chromatography, where lipids, even within the same class, have a large spread in retention time. Hence, analytes will differ in retention time from their internal standards, leading to standards not accounting for region specific effects such as ion suppression. Alternative chromatographic methods such as hydrophilic interaction liquid chromatography (HILIC) and supercritical fluid chromatography (SFC) can be used, where all lipids of a single class co-elute. Hence, semi-quantitation using appropriate correction factors to account for differences in ionization efficiencies based on carbon length and the number of unsaturation may be possible in HILIC and SFC, while in RP the use of standards for normalization should not be considered quantitative. Similarly, ion mobility may be applied to lipidomics, and since ion suppression occurs in-source before separation by ion mobility, lipid standards with varying fatty acyl-constituents from analytes may still be used to account for ion-suppression effects. In addition, collision cross section obtained from ion mobility can improve confidence in identifications, and ion mobility can be used to separate isomers, although in lipidomics there has been limited success as higher resolution separation by ion mobility is needed for lipids [9, 18–20].

In summary, the best choice of lipid internal standards are those that are lipid class representative and elute at similar retention times to the analytes of interest. Normalization by internal standards is important to reduce variation from sample handling and processing, data-acquisition, data-processing, and other sources which are not related to the study design. Reducing variance from these sources is simplified by the use of LMN, and may increase the detection of biomarkers and other differences between groupings. Manually selecting representative spiked internal standards and the associated lipid analytes to normalize and applying the algorithm for relative quantitation can be a tedious process prone to human error, especially with lists containing hundreds of lipid species. Automation of the quantification process can lead to increased throughput, a reduction in errors, and harmonization of quantification methods within the lipidomics community. Therefore, we developed LipidMatch Normalizer (LMN), which can be integrated in an open source workflow to select the most appropriate internal standards for relative quantitation within acquired LC-HRMS data. While numerous open source quantification software for direct-infusion based lipidomics currently exists [21–24], to our knowledge, Lipid Data Analyzer (LDA) [25, 26] is the only open source relative quantitation software for LC-based lipidomics using class representative lipid standards to return normalized values. LMN is unique to LDA and commercial lipid relative quantitation software such as LipidSearch (Thermo Scientific), SimLipid (PREMIER Biosoft), and Lipidyzer (SCIEX), in that it was built to be integrated into workflows using any combination of peak picking software (including the freely available software MZmine [27] and XCMS [28]) and peak annotation software. For example lipids can be normalized to internal standards by applying LMN to outputs from MS-DIAL [29], LipidSearch, and LipidMatch [30]. In addition, the LMN algorithm for selecting internal standards for feature quantification is unique; aiding in reducing ion suppression, matrix effects, and other chromatographic region specific effects by matching individual lipid species to lipid internal standards with the closest retention time and reducing the effect of structure related ionization efficiency differences by matching lipids to internal standards by lipid class and adduct. Because no absolute cutoff of retention time differences between standards and analytes are currently provided in LMN, in reverse phase chromatography chromatographic region specific effects may not be accounted for by internal standards differing substantially from analyte retention times.

As discussed, LC-MS based relative quantification has many more compounding factors influencing the choice of internal standards and the resulting values obtained than shotgun approaches, due to ion suppression effects being specific to elution time, lipid aggregation being enhanced during chromatographic purification of lipids, ionization efficiencies being based on mobile phase gradient, and carry-over. [10] While it is outside of the scope of this manuscript to comprehensively investigate all influences on the normalization values obtained, we investigate previously unstudied data-processing choices and the influences of these choices on normalized results. The effect of lipid structure on quantitation has been investigated previously [11, 16, 17, 31], while to our knowledge the effect of different data processing strategies and adducts utilized on final normalized lipid levels has not been examined thoroughly in UHPLC-HRMS experiments. Therefore, we investigated different data processing methods (peak area versus peak height, smoothing versus not smoothing) and utilization of different ions and polarities for lipid relative quantitation using LMN. Investigating the effect of various aspects of the lipidomics workflows on relative quantitation using open source tools available to the wider community is an important step in validating the utility and establishing community wide protocols for relative quantitation in lipidomics.

## Implementation

### Lipid extraction and data acquisition

Lipids were isolated from 40 μL of National Institute for Standards and Technology (NIST) standard reference material (SRM 1950) Metabolites in Frozen Human Plasma [32]. Lipid internal standards purchased from Avanti Lipids (Alabaster, AL), which included lysophosphatidylcholine (LPC(17:0)), phosphatidylcholine (PC(17:0/17:0)), phosphatidylglycerol (PG(17:0/17:0)), phosphatidylethanolamine (PE(17:0/17:0)), phosphatidylserine (PS(17:0/17:0)), triglyceride (TG(15:0/15:0/15:0)), ceramide (Cer(d18:1/17:0)), and sphingomyelin (SM(d18:1/17:0)), were spiked into the plasma at 1.4 nmol, 0.92 nmol, 0.93 nmol, 0.97 nmol, 0.92 nmol, 0.26 nmol, 1.3 nmol, and 0.98 nmol, respectively (resulting in final concentrations of 35 nmol/mL, 23 nmol/mL, 23.25 nmol/mL, 24.25 nmol/mL, 23 nmol/mL, 6.5 nmol/mL, 32.5 nmol/mL, and 24.5 nmol lipid/mL plasma). ^13^C_2_-cholesterol was purchased from Cambridge Isotope Laboratories (Tewksbury, MA), and spiked in at 1.8 nmol resulting in concentrations of 45 nmol lipid/mL plasma. The extraction was performed using the Matyash method [33] and samples were reconstituted in 200 μL of isopropanol.

Samples were injected onto a Waters (Milford, MA) BEH C18 UHPLC column (50 × 2.1 mm, 1.7 μm) held at 50 °C with mobile phase A consisting of acetonitrile:water (60:40, *v*/v) with 10 mM ammonium formate and 0.1% formic acid and mobile phase B consisting of isopropanol:acetonitrile:water (90:8:2) with 10 mM ammonium formate and 0.1% formic acid at a flow rate of 0.5 mL/min. A Dionex Ultimate 3000 RS UHLPC system (Thermo Scientific, San Jose, CA) coupled to a Thermo Q-Exactive mass spectrometer (San Jose, CA) was employed for data acquisition using both targeted and data-independent MS/MS acquisition for annotation. Mass spectrometric parameters and scan modes can be found in Additional file 1. The targeted MS/MS list can be found in Additional file 2.

### Data processing

**Fig. 1:**
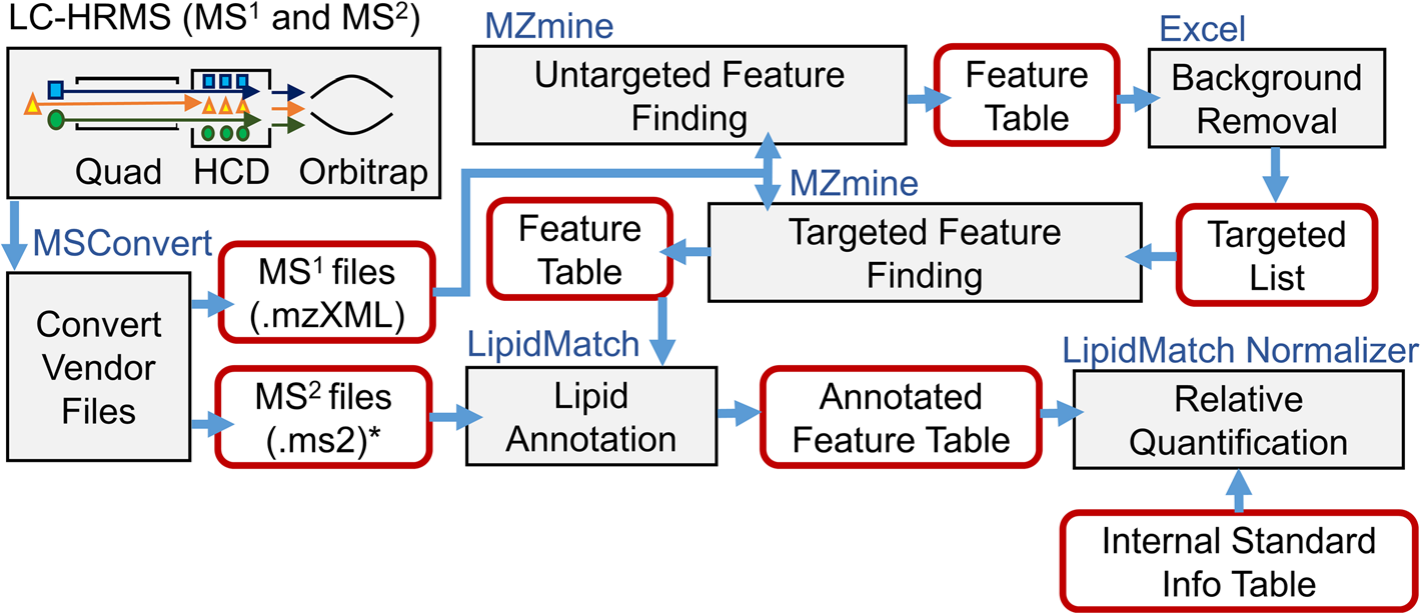
Open source lipidomics workflow employed in this study. Blue titles are software, grey boxes are processes, and red boxes are inputs/outputs. Note that both LipidMatch and LipidNormalizer are modular: LipidMatch can take in feature tables from any peak picking software, and LipidMatch Normalizer can normalize data from any identification software, allowing user flexibility. For more ideas and information on different workflows using these software see the following youtube video tutorials: https://www.youtube.com/playlist?list=PLZtU6nmcTb5mQWKYLJmULsfqNy9eCwy7K *for AIF both .ms1 and .ms2 files must be provided. Can handle data-dependent and targeted MS/MS data as well

The open source data processing workflow for lipidomics is shown in Fig. 1. The first step in the workflow is feature finding using MZmine 2 [27], followed by annotation with LipidMatch [30], blank feature filtering (BFF) [34], relative quantification by LipidMatch Normalizer (LMN), and reduction to molecular species using an in-house R script. All scripts and software are employed, and in-house scripts, LipidMatch, and LipidMatch Normalizer can be found at secim.ufl.edu/secim-tools/. Note that LMN can be employed with any feature finding and lipid identification software, and this is just one workflow in which it can be employed. More detailed description of the workflow can be found in Additional file 1.

### LMN user workflow

All steps prior to use of LMN, as well as the steps to use the LMN software are available as video tutorials which can be accessed at <https://www.youtube.com/playlist?list=PLZtU6nmcTb5mQWKYLJmULsfqNy9eCwy7K> and shown in Fig. 1. The LMN software requires two comma separated values (.csv) files as input for proper operation. The first required file is a feature table with the following content for each feature: (1) peak height or peak area, (2) lipid annotation, (3) lipid class, (4) lipid adduct, (5) retention time, and (6) *m/z*. Note this allows LMN to be compatible with any software which generate this information, including XCMS and MS-DIAL. The second required file is an internal standard sheet, which lists the names of all internal standards added, their concentrations, retention time, and *m/z* for each adduct. The names of the internal standards can be in any format familiar to the user. Examples and templates of the two input tables can be found in the LipidMatch Normalizer zip file available at <http://secim.ufl.edu/secim-tools/> and in the Additional file 3.

The user can easily generate the *m/z* of the adducts expected for each lipid internal standard using only the internal standard name, with a separate tool, LipidPioneer [35]. The user then specifies which internal standard will be used for each lipid class in the internal standard sheet. Note that multiple lipid classes can be represented by a single internal standard in the internal standard sheet. For example in this work, we included the following lipid classes to be normalized to PC(17:0/17:0): PC, Plasmanyl-PC, Plasmenyl-PC, and OxPC (oxidized phosphatidylcholine). We chose to represent ether-linked species using a non-ether-linked internal standard, as it has been shown that ether linked glycerophospholipids have the same response factor to their non-ether linked counterparts [31]. This internal standards sheet can be used for later experiments if the same internal standards and chromatographic conditions are employed (and there is no retention time drift).

After open and running the R script in the LipidMatch zip file, popup boxes prompt the user to select the working directory folder for all files (feature table and internal standards sheet). The user is then instructed to select the feature table and the internal standard sheet. The user completes a series of input boxes, entering the location of the columns for *m/z*, retention time, lipid class, lipid adduct in the feature table, and the row in which data starts. By not predefining the format of the feature table, users can utilize various peak picking and lipid annotation software and directly, or with minor modification, apply LMN. Other user inputs include retention time and *m/z* tolerances, which are used for locating features representing the internal standards in the feature table using the retention time and *m/z* values supplied in the internal standard sheet.

The software outputs a ‘standardsfound.csv’ (all identified internal standards) and ‘[input_sheet_name]_Quant.csv’ (feature table with normalized lipid levels and information on the internal standard used for each feature including standard rank) file. Lipids normalized using a ranking of 2 or 3, should be used only with great caution, as internal standards which match the lipid class of the feature were not found. Since lipid class significantly affects ionization efficiencies, these standards only take into account ion suppression, but not ionization efficiencies. An output table for LMN can be found in the LMN Additional file 3.

### LMN algorithm

**Fig. 2:**
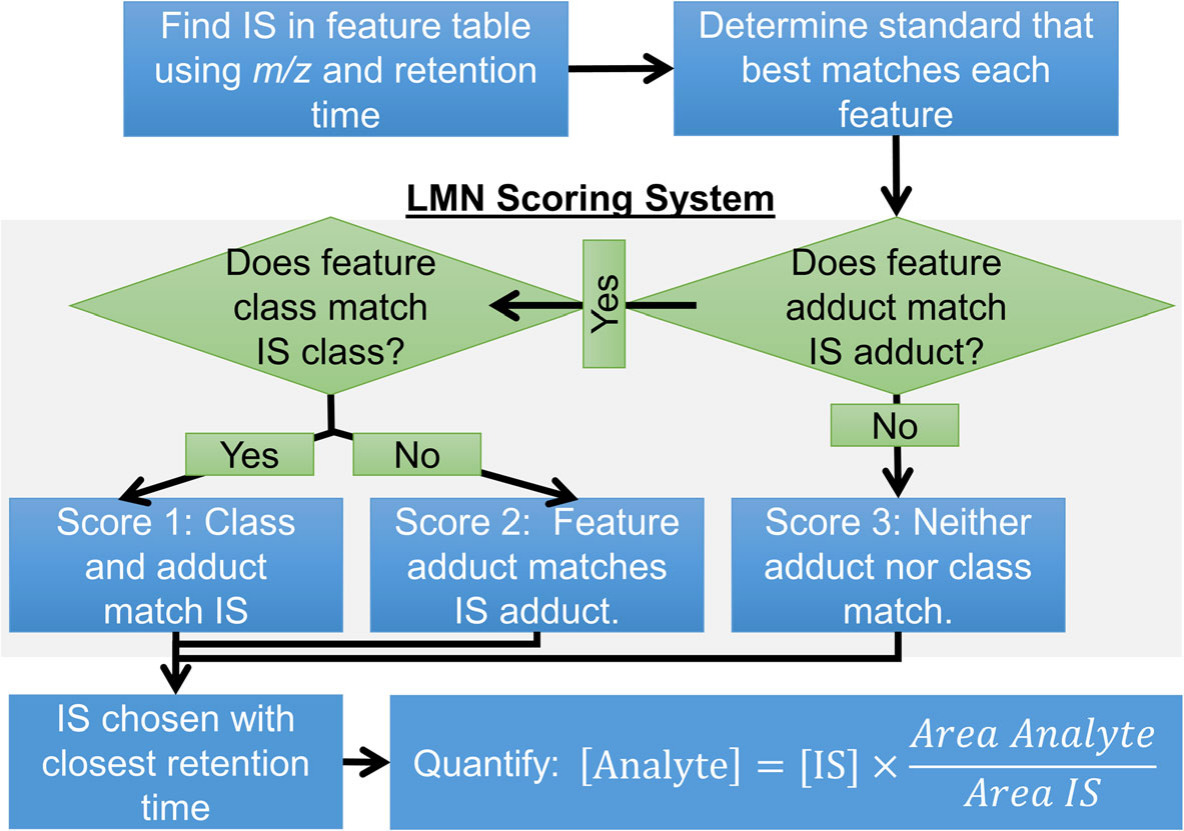
Simplified schematic of LipidMatch Normalizer (LMN) algorithm. The acronym IS stands for internal standard

LMN algorithms were validated for this dataset by manual relative quantification of all features. A schematic of the LMN algorithm is shown in Fig. 2. The LMN algorithm incorporates a ranking based approach to classify internal standards selected for each feature depending on how close they match the analyte of interest. A ranking represented by a small number indicates better representation of the feature by the internal standard while a ranking represented by a large number indicates poorer representation (with rankings of 1, 2, and 3). For each feature, the LMN algorithm associates the appropriate internal standard detected. If the feature and internal standard adduct and class match, the feature is ranked as a 1. If the current feature class does not match any of the internal standard lipid classes, but the same adduct is found for an internal standard representing a different lipid class, a rank of 2 is given. If no internal standard is found for a feature with a matching adduct or class, a rank of 3 is given (Fig. 2).

It is important to note that multiple internal standards can be provided for a single lipid class. In this case, the internal standard with the closest retention time is used for each feature of the respective lipid class. Since retention time correlates with saturation and carbons in the lipid fatty acyl chains, this will in part account for different ionization efficiencies due to these structural differences. More importantly, ion suppression can vary across retention time, and therefore using multiple internal standards can better account for these differences in ion suppression. If multiple standards are found using a rank of 2 or 3, the one with the closest retention time to the average retention time for the entire lipid class and specific adduct is used to normalize all lipids with the class and adduct.

### Comparison of quantitating using different data processing methods and different ions

Different data processing methods and ions were used for relative quantitation to determine which methods had the greatest effect on the precision of the final normalized values. The comparisons were: smoothing versus no smoothing (smoothing set to 15 in MZmine), peak height versus peak area, relative quantitation with negative versus positive ions, and quantitation on [M + Na]^+^ adducts versus the major precursor ion. The [M + Na]^+^ adducts were chosen because for the majority of lipids in positive ion mode an [M + Na]^+^ peak is present, and hence may affect relative quantitation through competitive ionization. For comparison of similarity, the slope and R^2^ of linear correlations on the log_10_ value obtained between the two comparative methods were used. In addition, Bland-Altman type plots [36] were used to determine the relative percent difference in concentrations using two different methods or ions for quantitation. A distinction was that instead of normalizing to the average, as is traditionally done for calculating percent difference to be visualized in Bland-Altman plots [37], the differences were normalized to the minimum values (hence giving a percent increase from the minimum value). When differences are normalized to the average, the absolute relative percent difference plotted against the fold change (fold changes greater than 1) is non-linear and asymptotic to 200%, while the relative percent difference, calculated by normalization to the minimum, is linear as compared to fold change and hence is easier to interpret (Additional file 1: Figure S1). The formula used to calculate relative percent difference is shown below:

$$ \mathrm{Formula}\ 1: Relative\ percent\ difference=\frac{x-y}{\min \left(x,y\right)}\times 100 $$

*Where x and y represent concentrations calculated using different methods or ions*

For comparison of overall deviation between measurements, the absolute value of x-y was taken in the formula above. In this case, if relative percent differences were at or below 50% using modified Formula 1, the results were considered similar (for example, 0.5 nmol/mL and 0.75 nmol/mL), while a relative percent difference above 50% was not considered similar (for example, 0.5 nmol/mL and any value greater than 0.75 nmol/mL). A sign test was used to determine whether the quantitative values using different methods or ions provided significantly similar results (less than or equal to 50% difference) across the majority of features or significantly different results (greater than 50% difference).

Precision of relative quantification using different methods or ions for replicate injections was determined using coefficient of variation (CV). A sign test was used to determine whether features tended to have higher CVs in one methodology compared to another.

## Results

### Comparison of targeted MS/MS versus AIF

A total of 129 unique lipid molecular species across 16 lipid types were identified in negative ion mode, of which 122 had appropriate internal standards for relative quantification (with phosphatidylinositols not having a class specific internal standard). In positive ion mode, 225 unique lipid molecular species across 20 lipid types were identified, with 185 normalized using appropriate class representative internal standards. A more detailed description of annotations, including a comparison of all-ion fragmentation (AIF) annotations with targeted MS/MS annotations can be found in Additional file 1. Briefly, Of the features annotated both by AIF and targeted MS/MS, 100% had the same annotation (top ranked, considering plasmenyl and plasmanyl species differing by one saturation the same) in negative ion mode, and 87% had the same annotation in positive ion mode. Of those in positive ion mode with differing annotations between AIF and targeted MS/MS, the annotations only differed by fatty acid composition, not by lipid class and total carbons and degrees of unsaturations.

### Comparison of different data-processing methods on normalized lipid levels

Different data processing methodologies and ions for normalization were compared in terms of final normalized lipid levels (normalized lipid levels can be found in the Additional file 3), as well as each method’s precision in measuring three replicate injections. The relative quantitation comparisons were as follows: (1) smoothed versus non-smoothed peak heights, (2) smoothed versus non-smoothed peak areas, (3) peak area versus peak height, (4) negative versus positive polarity (peak areas), and (5) major adducts versus sodium adducts (peak areas). The number of features used for each comparison, percent difference, and log two of the fold change, are summarized in Additional file 1: Table S3. For the comparison of different ions and polarities, only those lipid molecules which were represented by both ions, or both polarities, were used.

**Fig. 3:**
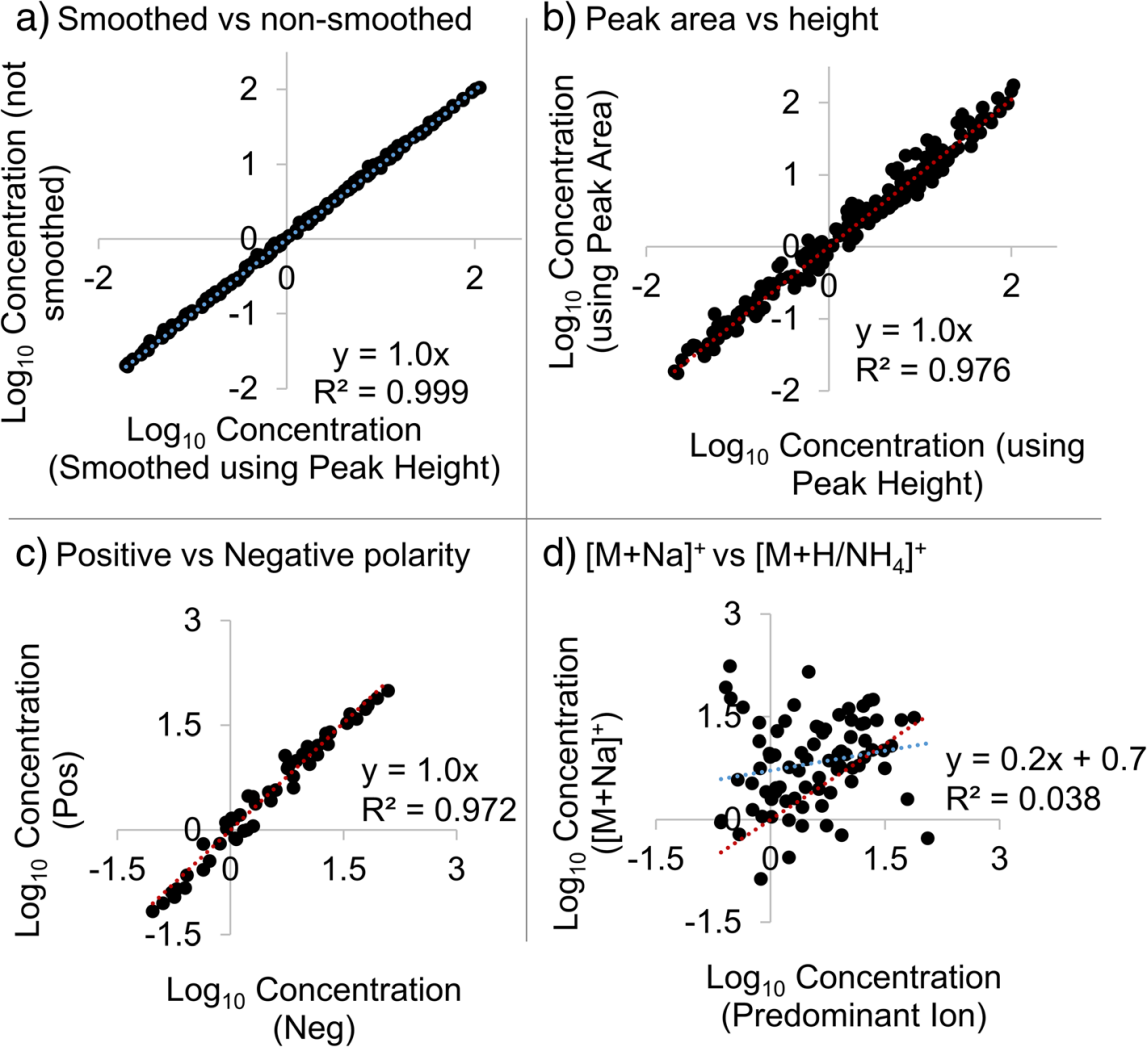
Linear regression comparing the log10 of normalized lipid levels calculated using different workflows and ions. A slope of 1 and R2 close to 1 are expected if the methods or ions both result in similar normalized lipid levels. The panels show normalized levels calculated using smoothed versus non-smoothed peak heights (smoothing was done as the final step in MZmine; *n* = 184; **a**), peak area versus peak height (*n* = 184; **b**), positive versus negative polarity using peak area (*n* = 51; **c**), and sodium adducts versus the major adduct observed in positive polarity using peak area (*n* = 76; **d**). For **d**, sodium adducts were compared to protonated adducts except in the case of neutral lipids which formed ammoniated adducts

**Fig. 4:**
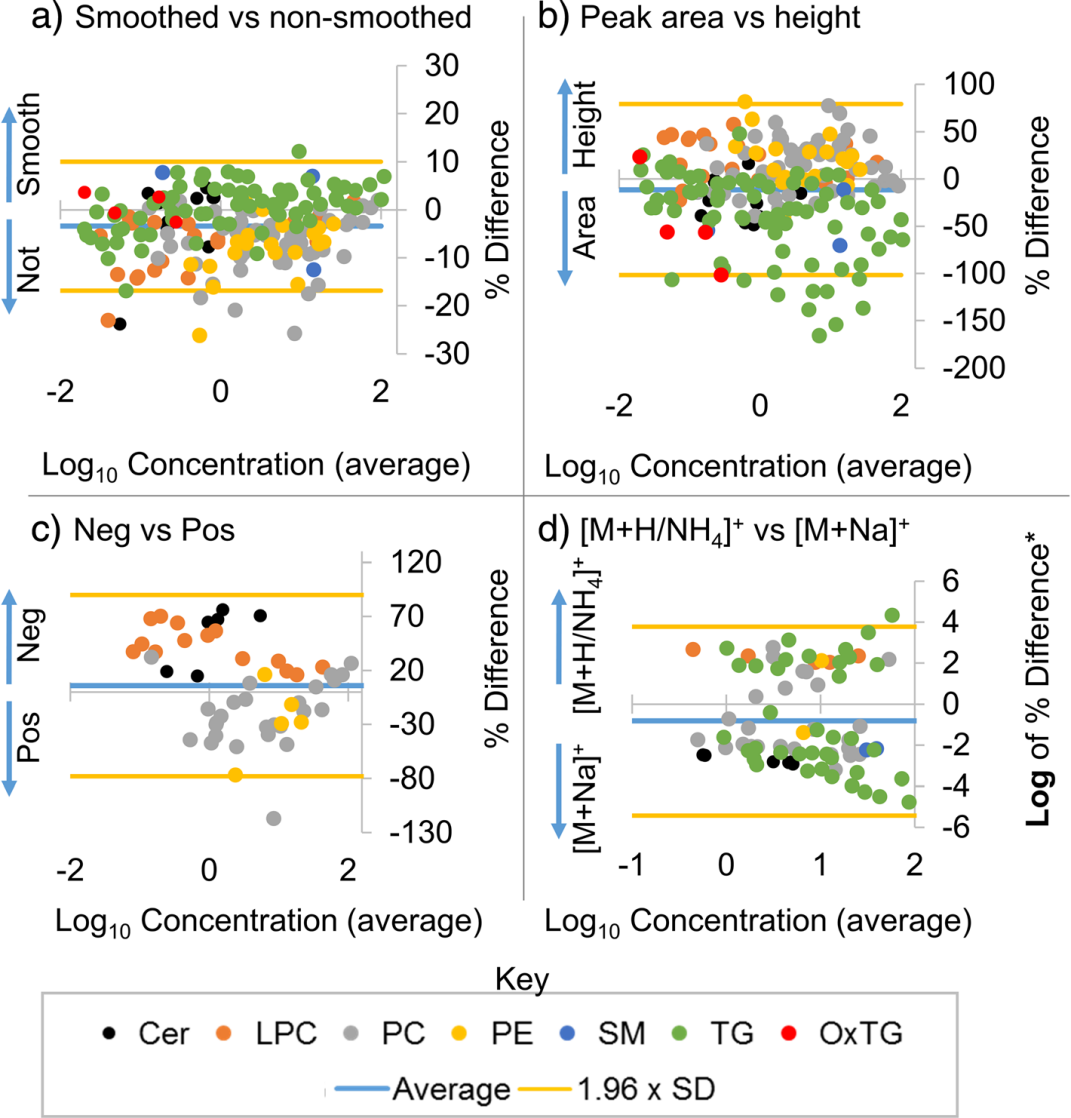
Bland-Altman type plots showing differences in normalized lipid levels calculated using different methods and ions. The panels show the percent differences in normalized lipid levels calculated using smoothed versus non-smoothed peak heights (smoothing was done as the final step in MZmine) (**a**), peak area versus peak height (**b**), positive versus negative polarity using peak area (**c**), and sodium adducts versus the major adduct observed in positive polarity using peak area (**d**). Note that orange lines represent 1.96 x standard deviation (the 95% limits), and hence are a measure of where you would expect 95% of the percent differences to fall for each comparison. See Formula 1 for relative percent difference calculation. Arrows delineate the direction of difference. *Note that the differences between major adducts and [M + Na]^+^ were drastic and ranged over several orders of magnitude. Therefore, the log of the absolute percent difference was used and then multiplied by − 1 when the [M + Na]^+^ normalized lipid level was calculated higher than the major ion

Comparisons of smoothed versus non-smoothed peak heights, peak area versus peak height, and normalization on positive versus negative ions, all had an R^2^ above 0.97 and slopes about equal to 1 in log-log plots shown in Fig. 3. Note that correlation is expected between two methods aimed at detecting the same concentrations, especially over wide ranges as in Fig. 3. [33] Hence, the correlation observed only suggests that the measurement methods were detecting the same phenomenon, not that they provided the same result. But modified Bland-Altman plots and sign tests confirmed that the three methods provided comparable normalized lipid levels. A significant proportion of relative percent differences were at or lower than 50% for comparisons (Fig. 4), with *p*-values of a two-sided sign test less than *p* < 0.05. Smoothing had the least impact on normalized lipid levels, with none of the 185 lipids above 50% difference, and only two above 25% difference. Peak height versus peak area also provided relatively similar normalized lipid levels with only about 13% of the 185 lipids above 50% difference. Of these three comparisons, polarity had the greatest effect on normalized lipid levels, with 25% of lipids having percent differences above 50% (in this case only the 51 lipids common between polarities were utilized (Additional file 1: Table S3d).

### Precision of measurements using different data-processing strategies

For all methods, the average CV of normalized lipid levels was less than 20% (Table 1). Normalized lipid levels calculated using positive polarity, peak area, and non-smoothed data were more reproducible across multiple injections when compared to normalized lipid levels calculated using negative polarity, peak height, and smoothed data, respectively, as indicated by a two-tailed sign test and lower CVs (Table 1). Note that for the higher CV in negative ion mode, results could be due to an increased injection volume in one of the negative ion mode samples.

**Table 1:** Comparison of the coefficient of variation (CV) of normalized lipid levels in three replicate injections calculated using different methods or ions

Test	CV (Avg)	CV (# >)^a^	Sign Test
[M + H/NH4]^+^	5 ± 3%	31	*p* = 0.057
[M + Na]^+^	10 ± 10%	49	
Pos	4 ± 5%	10	*p* < 0.0001
Neg	12 ± 15%	42	
Height	7 ± 5%	126	*p* < 0.0001
Area	6 ± 7%	59	
Smoothed	7 ± 6%	103	*p* < 0.0001
Not Smoothed	6 ± 5%	82	

^a^The number of species with CVs greater in the respective method or ion

Note that comparison for ions were made using peak areas, while those for smooth versus not smoothed utilized peak heights. Note that negative ion mode had an injection with a different volume than the remaining injections, and hence this could be the reason for increased CV as compared to positive ion mode

In addition to the comparisons between each method, CV was compared before and after normalization, to determine if normalization to internal standards using LipidMatch Normalizer reduced variation in replicate injections. In positive ion mode the average % CV was nearly 2-fold higher prior to normalization at 10 ± 7% versus 6 ± 7% after normalization, with differences significant based on a student t-test (*p*-value = 0.00000001). In negative ion mode, the differences were much more pronounced, due to an increased injection volume in one of the samples, which was at least partly accounted for during normalization. The average % CV in negative ion mode was 71 ± 19% prior to normalization, and 14 ± 17% after normalization.

## Discussion

### Software features compared to other relative quantification software

Available lipid quantitation software which can process data from UHPLC-HRMS/MS workflows are compared in Table 2. To our knowledge, LMN and LDA are the only software programs for LC-HRMS/MS data which are both open-source and can employ class representative relative quantitation using internal standards. While LDA is a full solution, from feature detection to quantitation, LMN can more easily be integrated into workflows, leveraging other open source tools, for example MZmine and LipidMatch, as employed in this manuscript. Peak picking and lipid annotation can be performed with various software, and parameter optimization can be application, instrument, and workflow specific. Therefore, by integrating LMN into a larger open source or proprietary lipidomics workflow, users do not need to validate and optimize new peak picking and annotation strategies. The only requirements are a separate column in the feature table for lipid retention time, *m/z*, class, and adduct. This can be obtained using the text to columns function in Excel if the information is not separated in the native output format. Aspects of the lipidomics workflow, including peak picking and identification of lipids, can take hours to days for even small sample sizes (e.g. 10). Relative quantification of thousands of lipids across large sample sizes (e.g. hundreds) using LMN and other open source software have total run times on the order of seconds to minutes and therefore computational time is not of concern.

**Table 2:** Comparison of different lipid quantification software which can be applied to UHPLC-HRMS/MS data

	Output	IS: Class Specific^a^	Multiple IS per Class^b^	Response Factors^c^	Vendor Specific	License	Modular^d^
Lipid Data Analyzer	Concentration^e^	Yes	Yes	No	No	Open Source	No
MZmine 2	Normalized Peak Intensities	No	_	No	No	Open Source	No
LipidMatch Normalizer	Concentration^e^	Yes	Yes	No	No	Open Source	Yes
SimLipid	Concentration^e^	Yes	Yes	No	No	Purchase	No
LipidSearch	Concentration^e^	Yes	No	No	No	Purchase	No

^a^Can internal standard be matched to features for quantification based on lipid class?

^b^Can multiple internal standards for a single lipid class be used?

^c^Are response factors based on lipid structures and resulting ionization efficiencies employed?

^d^Can the tool be used with various feature finding and identification software?

^e^Note that for these software while outputs are technically in units of concentration, they should not be interpreted as quantitative, but rather as normalized abundances to class representative internal standards (relative quantification)

### Annotation using LipidMatch and AIF data provides accurate annotations

Prior to reconstruction of precursor-fragment relationships using LipidMatch algorithms or similar, AIF proves to be high in false positives. Results show that LipidMatch algorithms for annotation using AIF provided the same results to targeted and data-dependent MS/MS methods, without increased false positives at the level of lipid class, total carbons, and degrees of unsaturation.

### Comparison of normalized values across studies highlight that generally lipidomics is not quantitative

The final normalized lipid levels were compared to both the NIST inter-laboratory study [12] and the LIPID MAPS consortium analysis of NIST SRM 1950 [38]. The values diverged significantly between all three studies for lipids summed at the level of carbons and double bonds (Additional file 4: Tables S4 and S5). These results emphasize that single point calibration using class representative internal standards in reverse phase is a normalization method and not quantitative. Hence, the advantages of internal standard based normalization are a reduction in variance of measurements and better statistics as discussed in the prior paragraph, but values are not absolute amounts which can be comparable across laboratories and techniques. But other approaches using LMN could be considered semi-quantitative. Because standards and analytes co-elute in separation techniques such as SFC and HILIC (because all species within a lipid class co-elute), the application of LipidMatch Normalizer along with appropriate correction factors for ionization efficiencies could be semi-quantitative. In the case of SFC or HILIC separation, equivalent carbon number should be used instead of retention time to match standards with analytes of similar ionization efficiencies.

### Data-processing methods used affect the accuracy of lipid levels measured

Polarity was shown to have the second greatest effect on resulting normalized lipid levels. This has major implications for which polarity is chosen as “correct” for a given set of lipids. Often the feature with greater peak areas or heights is chosen, which would always favor positive ion mode. On the other hand, negative ion mode has lower background signal, signal to noise, and, for glycerophospholipids, more accurate identification.

**Fig. 5:**
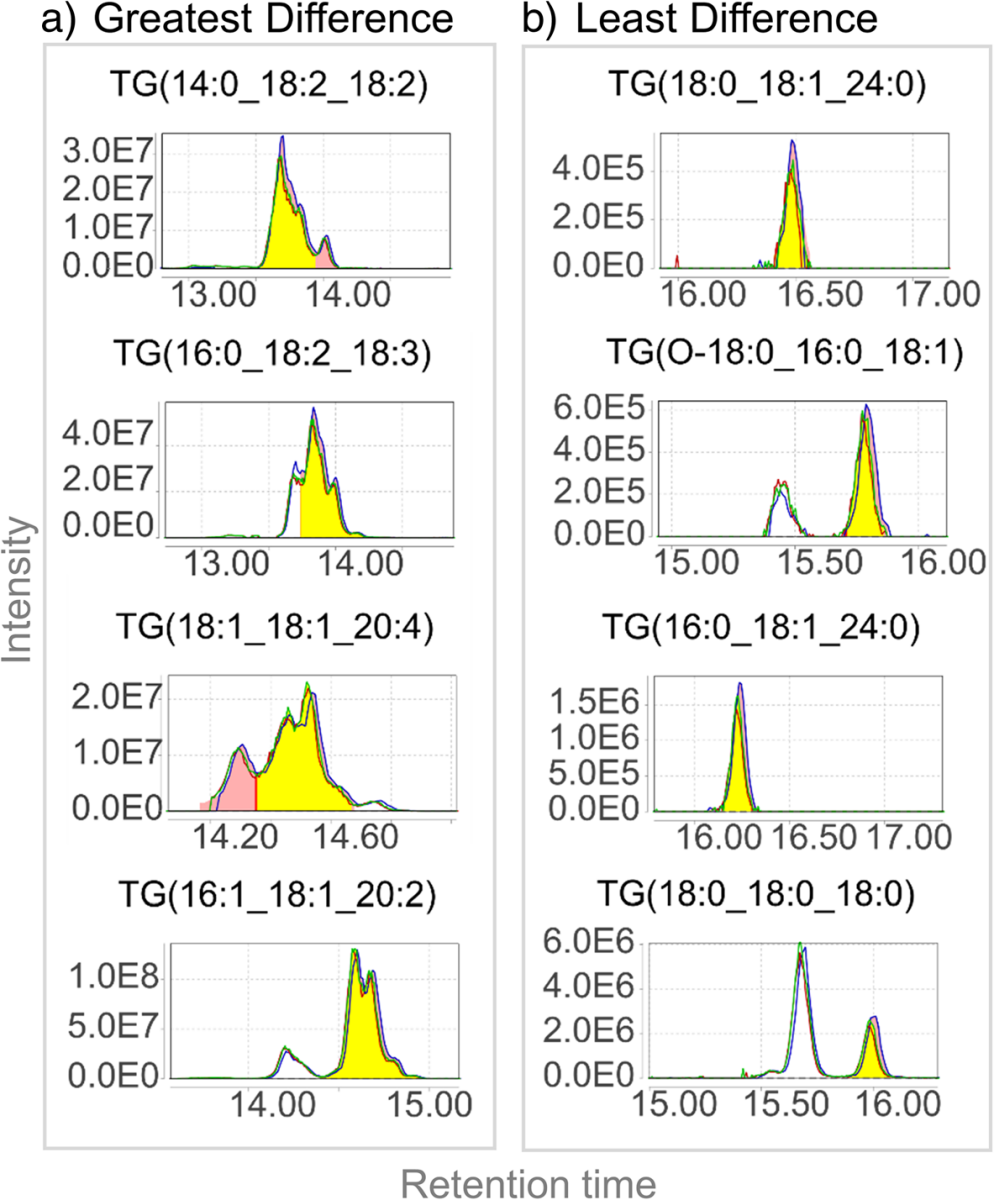
Extracted ion chromatograms (EICs) and peak integration by MZmine of the triglycerides (TGs) with the most (**a**) and least (**b**) percent difference when comparing quantitation using peak height versus peak area

Peak area versus peak height had the third greatest, although minimal, impact on resulting normalized lipid levels. For comparisons of peak area versus height, the greatest percent difference was for triglycerides, with normalized lipid levels calculated in peak area much greater than those calculated by peak height. For 10 of the 59 triglycerides, the normalized lipid levels calculated using peak area were more than 2-fold higher than those calculated by peak height (over 100% percent difference; Fig. 4b). A closer look at extracted ion chromatograms (EICs) and integration using MZmine 2 of these peaks showed a common trend (Fig. 5).

Triglyceride isomers are notoriously difficult to separate, due to the numerous possible combinations of the three fatty acids which lead to the same number of carbons and double bonds, with resulting isomers having the same or similar retention behavior. For the triglycerides with minimal difference between peak height and peak area (less than 5% in Fig. 5b and Additional file 1: Figure S4b), the peaks were well defined (Gaussian shaped and baseline resolved) without any visual overlap. For the triglycerides with major differences between peak height and peak area (over 100% in Fig. 5a and Additional file 1: Figure S4a), there were overlapping isomers without complete deconvolution. Therefore, the integration of multiple overlapping isomers as one peak (improper deconvolution and/or poor chromatographic separation) was the major cause explaining why normalized lipid levels calculated using peak areas were much greater than those using peak height. In addition, the number of isomers integrated as one peak varied across samples (Fig. 5a and Additional file 1: Figure S4a). This led to a large variation in normalized lipid levels calculated using peak areas in the case of overlapping peaks, and hence using peak height in lipidomics may be advantageous when a large portion of isomeric peaks overlap in retention time.

The majority of lipid normalized lipid levels calculated in positive and negative polarity differed by less than 50%. For those which differed by more than 50%, there was no clear trend in extracted ion chromatograms (EICs). For example, the EICs of PC(16:0_20:5) and PC(18:0_20:4) had similar elution profiles between species and as protonated and formate ions (Additional file 1: Figure S5). While EICs looked similar, normalized lipid levels calculated in negative and positive polarity for PC(16:0_20:5) differed by over 2-fold (over 100%), while for PC(18:0_20:4) normalized lipid levels differed by less than 10%. This data suggest that certain species may have very different ionization efficiencies compared to the internal standard and response curves for negative and positive polarity, while others do not. Indeed, Zacarias et al. [31] showed non-linearity in intensity versus normalized lipid level in negative ion mode irrespective of instrumental parameters, while lipid intensity versus normalized lipid level in positive ion mode was relatively linear in comparison.

While adducts determined in negative ion polarity correlated well and gave similar normalized values as adducts in positive polarity, sodiated adducts gave very different normalized lipid levels (Fig. 4d) and did not correlate with their corresponding adducts in positive polarity (Fig. 3d). For comparison of relative quantitation using major ions versus sodium ions, a targeted list for sodium was developed by copying retention times and changing the masses of the [M + H]^+^ and [M + NH_4_]^+^ ions detected. This conversion of protonated and ammoniated species to a sodiated *m/z* was automated by pasting the molecular species into LipidPioneer [35]. The targeted peak list was then uploaded and the data were reprocessed using MZmine as described in the methods section. No trends were observed in the peak heights or areas of the sodiated species and their corresponding adducts ([M + H]^+^ or [M + NH_4_]^+^) (Fig. 3d), suggesting that a completely different phenomena was controlling ion signal measured in sodium versus other adduct species. [33] This is potentially due to sodium not being added to solution, and hence concentrations of sodiated species could be impacted by the number of sodium ions dissolved in the mobile phase at the point of elution, the number of competing ions forming sodiated species, co-eluting isomers, the amount of sodium in the matrix and the concentration of the analyte. As shown by lack of correlation to major adducts, the concentration of analyte seems to be a minimal factor in the intensity of sodium adducts of the analyte. It is possible that adding signal intensities of all adducts for the same molecular species and the associated standard could improve relative quantitation by improving the amount of signal used for a given ion and reducing variance from competitive ionization between adducts, although this was not observed. When adding [M + Na]^+^ to [M + H]^+^, there was a slight increase in the relative percent difference between the normalized lipid levels calculated in positive ion mode compared to negative ion mode for LPCs and PCs and a significant decrease in the percent difference for ceramides. But due to the instability of the sodium adducts intensities across injections, it is not recommended to perform relative quantification using sodium adducts (unless summed with all other adduct intensities).

### Lipid normalization using LMN increases precision of measurements

In certain cases, precision in calculating lipid levels is more important than accuracy, in order to reduce measurement variance and increase the likelihood of observing changes across sample groups. The use of LMN and lipid normalization reduced variance in both positive and negative ion mode, as compared to non-normalized lipid values. The results in negative ion mode shows that normalization to lipid internal standards reduces variance from minor or major differences in injection volume. Therefore the use of LMN and normalization to a single point calibration in lipidomics may reduce variance from instrumental, experimental, and other sources which are not related to the study design, and increase the potential of discovering changes across sample groups.

In addition it was found that for replicate analyses of a sample, the CV varied depending on data-processing strategies. Generally the use of peak area and non-smoothed samples had less variance than the use of peak height and smoothing. These results may not be generalizable to all datasets and workflows, and further experiments should be done comparing the effect of these parameters on CV.

## Conclusions

LipidMatch Normalizer (LMN) employs internal standards to normalize lipids in UHPLC-HRMS/MS open source workflows, including both in reverse phase and in HILIC and SFC (using equivalent carbon number, rather than retention time, for matching standards to analytes for SFC and HILIC). The flexibility in the input feature table format allows LMN to be used as a backend to any lipid annotation software. LMN utilizes a unique algorithm to select a standard to normalize the lipid analyte by matching lipid class, adduct, and retention between the feature and the internal standard in order of priority, respectively. LMN allows for multiple internal standards per lipid class and provides a ranking system allowing for transparency, noting how each internal standard was chosen for each lipid class and adduct. The percent CV across replicate injections was found to be significantly reduced in both positive and negative ion mode when applying LMN.

Applying LMN to compare normalized values obtained using various data processing workflows and ions, we found that the ion chosen for normalization had the greatest effect on the resulting relative quantification. Negative and positive ions showed slightly different normalized lipid levels, while sodium ions provided drastically different lipid levels compared to all other ions. We suggest not to utilize sodium adducts in calculating lipid concentration, at least in cases where sodium is not intentionally added to the mobile phase and samples. Data processing had less of a significant effect, with the greatest difference in calculated normalized lipid levels being attributed to peak area versus peak height, when the feature consisted of multiple unresolved chromatographic peaks.

Additional features which could be employed for relative quantification, include response factors based on instrument response to lipid structure (carbons and degrees of unsaturation), and dialogue boxes to aid users in selecting internal standards when class representative standards do not exist. Our solution provides automation for studies where differences between groups, but not absolute quantification, is of interest. It is important to note that ion suppression and lipid aggregation effects on the resulting normalized values are not well understood, and hence fundamental studies on these effects are needed to optimize and validate relative quantification strategies for LC-MS. [10] Therefore, it is important to limit differences between internal standards used and analytes normalized. Users should design experiments carefully to choose internal standards which are exogenous to their sample (without any overlap in mass and retention time), best represent the analytes to be quantified, and are spiked in concentrations similar to the lipids being quantified.

A suite of new scripts have been introduced which can be used alongside LMN for lipid feature finding, filtering, identification and combining polarities and adducts. These are modular, and hence researchers can design their own workflow to meet their needs. In addition, LipidMatch Flow is available as a beta version, and combines all portions of the workflow into a single user interface. All scripts and video tutorials can be found at: < http://secim.ufl.edu/secim-tools/>.

## Availability and requirements

Project name: SECIM tools

Project home page: The current version is available at:

http://secim.ufl.edu/secim-tools/

Operating systems: Most operating systems (tested on Windows XP, Windows 7, Windows 10, Mac OSX)

Programming language: R (version 3.3.3)

Other requirements: The R Project for Statistical

Computing, Version R 3.3.3, https://cran.r-project.org/bin/windows/base/old/3.3.3/

License: GNU GENERAL PUBLIC LICENSE Version 3, 29 June 2007

Any restrictions to use by non-academics: no restrictions

## Additional files

Additional file 1: Contains **Figure S1** through **Figure S5**, and **Table S1** through **Table S3**. (DOCX 941 kb)

Additional file 2: Inclusion List for targeted MS/MS. Contains a Thermo formatted inclusion list for targeted MS/MS. (XLSX 19 kb)

Additional file 3: LipidMatch Normalizer Software. The LMN_Software.zip file contains batch files for lipidomics with MZmine processing and the LipidMatch Normalizer R script. The .zip file also contains files to guide the user in using LipidMatch, which include: A manual and troubleshooting document, and example input and output data (the data used in this paper). For the most up to date version of LipidMatch Normalizer please visit: http://secim.ufl.edu/secim-tools/. (ZIP 126 kb)

Additional file 4: LipidMaps, NIST interlab, and this studies lipid values for NIST SRM 1950. An excel table with the lipids identified in the LIPID MAPS, NIST Interlaboratory Study for Lipidomics, and this study for NIST SRM 1950, and the resulting lipid levels. (XLSX 23 kb)

## Abbreviations

AIF: All ion fragmentation
BEH: Ethylene bridged hybrid
BFF: Blank feature filtering
Cer: Ceramide
csv: Comma separated values
CV: Coefficient of variation (also termed % RSD or percent residual standard deviation)
EIC: Extracted ion chromatogram
ESI: Electrospray ionization
ether-LPC: Plasmenyl and plasmanyl lysophosphatidylcholine
ether-PC: Plasmenyl and plasmanyl phosphatidylcholine
ether-PE: Plasmenyl and plasmanyl Phosphatidylethanolamine
ether-TG: Plasmenyl and plasmanyl triglyceride
HRMS: High-resolution mass spectrometry
LDA: Lipid Data Analyzer
LIPID MAPS: Lipid Metabolites and Pathway Strategies
LMN: LipidMatch Normalizer
LPC: Lysophosphatidylcholine
*m/z*: Mass to charge ratio
MS/MS: Tandem mass spectrometry
NIST: National Institute of Standards and Technology
OxPC: Oxidized phosphatidylcholine
OxTG: Oxidized triglyceride
PC: Phosphatidylcholine
PE: Phosphatidylethanolamine
PG: Phosphatidylglycerol
PI: Phosphatidylinisitol
SM: Sphingomylin
SRM: Standard reference material
TG: Triglyceride
UHPLC: Ultra-high performance liquid chromatography
