# Supplementary material for: Software tool for internal standard based normalization of lipids, and effect of data-processing strategies on resulting values
Source: BMC Bioinformatics. 2019 Apr 29;20:217. doi: 10.1186/s12859-019-2803-8 (PMC6489209; doi:10.1186/s12859-019-2803-8)
Supplement: Supplementary file 1 — Contains Figure S1 through Figure S5, and Table S1 through Table S3. (DOCX 941 kb) [file 12859_2019_2803_MOESM1_ESM.docx]

Supplemental Information

**Influence of Data-Processing Strategies on Normalized Lipid Levels using an Open-Source LC-HRMS/MS Lipidomics Workflow**

## Jeremy Paul Koelmel, PhD; Jason A. Cochran, High School; Candice Z. Ulmer, PhD; Allison J. Levy, BS; Rainey E. Patterson, PhD; Berkley C. Olsen, BS; Richard A. Yost, PhD; John A. Bowden, PhD; Timothy J. Garrett, PhD

Author Emails:

Jeremy Koelmel <jeremykoelmel@gmail.com>

Jason Cochran <aceman905671@ufl.edu>

Candice Ulmer <czulmer@gmail.com>

Allison Levy <allisonjlevy@chem.ufl.edu>

Rainey Patterson <rpgarland@eastman.com>

Berkley Olsen <berkley0@phhp.ufl.edu>

Richard Yost <ryost@chem.ufl.edu>

John Bowden <john.bowden@ufl.edu>

Timothy Garrett <tgarrett@ufl.edu>

**Table of Contents:**

| *Additional Text:* Detailed Methodologies | pg 2 |
| --- | --- |
| *Additional Text:* Annotations and AIF | pg 6 |
| *Additional Text:* Interlab and LipidMaps Comparison | pg 10 |
| *Figures S1 – S5* | pg 12 |
| *Table S1 – S3* | pg 17 |

Note: The LipidMatch Normalizer (LMN) manual, software and tutorial videos can be found at:

SECIM.UFL.EDU/SECIM-TOOLS/

*Methods: Mass Spectrometric Parameters and Scan Modes*

Three replicate injections were run in both positive and negative polarity employing alternating full and all-ion fragmentation (AIF) scans at a resolution of 70,000 (FWHM) at m/z 200. In addition, 15 injections employing targeted MS/MS data acquisition were performed, with the inclusion list (supplemental file Supplemental_Targeted_List.xlsx) for fragmentation containing lipids identified by the LIPID MAPS consortium. The ion optic settings for the mass spectrometer included: analyzer temperature of 30 °C and S-Lens radio frequency level of 35 V. The ionization conditions included a sheath gas flow of 30, auxiliary gas flow of 5, and sweep gas flow of 1 arbitrary units, and a spray voltage of 3.5 kV, and capillary temperature of 250 °C. For positive ion mode, lock masses of diisooctyl phthalate (m/z 391.2842) and polysiloxanes (m/z 371.1012 and 445.1200) were used, while no lock masses were used in negative ion mode. Both AIF and targeted MS/MS injections were used for lipid identification, while full scan data were used for feature finding and relative quantification. The UHPLC gradient use in this experiment is shown in Table S1, while the mass spectrometric parameters are shown in Table S2.

*Methods: Lipidomics Workflow*

A two-step process was used for feature detection. First features and their respective *m/z*, retention time, and peak heights across samples were detected using an MZmine workflow consisting of mass detection, chromatogram building, chromatogram deconvolution using local minimum, isotopic peak grouping, and alignment and gap filling of features (the batch mode is in the supplemental 2017_9_11_LMN_Software.zip file; 2017_8_01_MZMine_Batch_Step1.xml). This step is untargeted, in that no information on expected peaks is utilized. In this work, the untargeted step for feature detection included a solvent blank and three replicate injections of SRM 1950 in both positive and negative polarity. The resulting feature table from the untargeted step was filtered using a modified blank feature filtering (BFF) [1] approach, where the minimum intensity of the replicate injections had to be at least five fold greater than the blank intensity. The BFF method dramatically reduces the number of peaks which are not from biological origin, and has been shown to better detect true positives and negatives as compared to other filtering approaches.[1]

After filtering, the median peak height and peak retention time from the SRM 1950 replicate injections were used to develop a targeted peak list. In the second step, a targeted list of peak *m/z* and retention time values was generated from the previous step, and the internal standard *m/z* values and retention time values were appended to this list. An MZmine workflow consisting of mass detection, targeted peak detection, chromatogram deconvolution, alignment, and gap filling was used (the batch mode is in the supplemental 2017_9_11_LMN_Software.zip file; 2017_8_01_MZMine_Batch_Step2.xml).

Reprocessing the data using a targeted peak list determined from a smaller sample set has two advantages, especially for application to larger datasets. One advantage is that this workflow significantly reduces data processing time for large datasets, while the other advantage is that peak picking and integration using a targeted peak list is more consistent across samples than aligning features from an untargeted workflow. For example, if there are six pooled samples which should be representative of the features present in 100 samples, these pooled samples are the only samples that need to be run through the initial MZmine workflow. Then a target list can be generated after blank filtration and subsequently used to target features across all 100 samples. Note that in this study only three samples were analyzed, and hence all samples were used to determine the targeted peak list. The median of retention time and *m/z* values across all samples was used rather than the average, as often overlapping peaks lead to average *m/z* and retention time values which are actually between the two peaks and neither represents the first or second peak. For cases in which there are odd sample numbers, the median will always represent the location of a true peak. For cases in which there are even sample numbers, the median will represent the average of two peaks and therefore the value at the i^th^ position of the ranked values can be used, where i = n/2, and n is the total number of samples.

Once the final peak list with retention time, *m/z*, peak area, and peak heights were obtained, the data were annotated using LipidMatch (Figure 1). LipidMatch [2] identification was performed using all ion fragmentation data (AIF) and targeted MS/MS data acquisition using precursor ions from lipids determined by the LIPID MAPS consortium [3]. If multiple lipids are annotated for a single feature, the lipid annotations are then ranked by the sum of fragment intensity.

The annotated feature table was further reduced to molecular species in positive and negative ion mode using a new R script available at <secim.ufl.edu/secim-tools/> in the LipidMatch additional tools folder and in the supplemental information (2017_9_11_LMN_Software.zip). The algorithm selects the top most abundant ion excluding sodium (for reasons discussed in the Results and Discussion section) and can also be used to combine negative and positive polarity datasets (preferentially choosing negative ions over positive ions of the same lipid molecule due to lower background and more accurate identifications in negative ion mode).

The finalized feature tables in negative and positive ion polarity with unique lipid molecular species was uploaded into LipidMatch Normalizer (LMN) for relative quantification. A table containing the internal standard name and corresponding concentrations (nmol lipid per mL plasma) was created and uploaded.

*Results and Discussion:*

*Lipid annotation, coverage, and comparison of normalized values to other studies*

A total of 129 unique lipid molecular species across 16 lipid types were identified in negative ion mode, of which 122 had appropriate internal standards for relative quantification (with phosphatidylinositols not having a class specific internal standard). In positive ion mode, 225 unique lipid molecular species across 20 lipid types were identified (the majority of ions ionize more efficiently in positive ion mode as compared to negative ion mode), with 185 normalized using appropriate class representative internal standards. Lipid types were defined as lipid classes as described in LipidMaps [4], although chain modifications and linkages (for example, ether-lipids and oxidations) were considered a single lipid type, no matter how many occurrences were observed across different lipid classes. The output tables with concentrations calculated for SRM 1950 data acquired in positive and negative mode using LMN and peak areas can be found in the supplementary LMN_Software.zip file under Example_Files. Note that the number of identifications in the example files refer to all lipid ions (including multiple adducts representing a single lipid molecules), and hence is higher than the number of unique lipids described above. These outputs are the .csv files generated via LMN, and include the LMN normalization rank, and the internal standard species and adduct used for normalization for each feature. Annotations in column 9 of the tables were obtained from LipidMatch, with an annotation beginning with "1_" representing identifications by targeted MS/MS, and "2_" by AIF. The majority of annotations were obtained using all ion fragmentation (AIF), with the remainder identified using targeted MS/MS.

In AIF, the precursor-fragment relationship is lost due to the wide isolation window, which allows all ions within the *m/z* range of interest to be fragmented. This can lead to a drastic increase in false positives as fatty acid fragments and class specific fragments observed from various non-isobaric species may be used to confirm the identification of a non-existent species. LipidMatch filters fragments using a correlation cutoff (with a user-modifiable default of 0.6) obtained from a linear regression of the elution profile of the precursor against the fragment ions. This AIF algorithm is advantageous in correctly annotating closely eluting peaks, as compared to using data-dependent scans (Supplemental Figure S2). Due to the high number of fragmentation scans in AIF for any precursor, the elution profile of the reconstructed mass chromatogram of the fragment specific to one overlapping isomer, but not the other, can be used to annotate the closely eluting peaks (Figure S2). On the contrary, in data-dependent approaches, only a single scan is often obtained across two overlapping elution profiles of isomers, and therefore it can be difficult to discern which chromatographic peak belongs to which isomer.

Example elution profiles of precursors and respective fragments are shown in Figure S3 for PC(16:0_20:4) and PC(18:2_20:4) identified in positive and negative ion mode, respectively. For PC(16:0_20:4) all precursor and fragment peaks elute with a similar profile at 7.6 minutes, while for PC(18:2_20:4) all precursors and fragments elute with a similar profile at 7.1 minutes. Overlapping elution profiles in the AIF reconstructed mass chromatograms are due to numerous lipids of different precursor mass containing the same fatty acyl constituents. For example, note that the shared arachidonic acid (20:4) leads to the same fragmentation elution profile for NL R_1_COOH, LPC(R_2_)+H, and R_2_COO^-^ in Figure S3. This indicates why it is important to employ correlation of elution profiles of precursors and fragments in AIF to reconstruct the precursor-fragment relationship, rather than only identifying lipids based on the occurrence of their respective fragments in the retention time region they elute. Ideally, if employing AIF, targeted MS/MS or data-dependent MS/MS is also employed (for example on a pooled sample) to help confirm annotations and increase coverage. One of the limitations of AIF is an increase in false negatives because lipid ions may not meet correlation thresholds required for identification if there is an overlap in fatty acyl or head group fragments in the MS/MS spectra. This overlap occurs often due to the shared moieties across different lipid types and within lipid types. Greater separation achieved in chromatography (or using other techniques in combination or separately such as ion mobility) can limit this overlap in shared fragment ion, and hence reduce false negatives in AIF.

Of all lipids identified in negative ion mode, 98 features were uniquely identified by AIF, 20 uniquely identified using targeted MS/MS, and 11 identified by both AIF and targeted MS/MS. In positive ion mode, 85 features were uniquely identified by AIF, 88 by targeted MS/MS, and 52 by both. Of the features annotated both by AIF and targeted MS/MS, 100 % had the same annotation (top ranked, considering plasmenyl and plasmanyl species differing by one saturation the same) in negative ion mode, and 87 % had the same annotation in positive ion mode. Of those in positive ion mode with differing annotations between AIF and targeted MS/MS, the annotations only differed by fatty acid composition, not by lipid class and total carbons and degrees of unsaturations. The majority (6 of 7) of the features with differing annotations using AIF versus targeted MS/MS were annotated as TGs, which are known to be difficult to annotate due to significant overlap of isomers in the retention time regime. These results suggest that annotation of AIF data using LipidMatch provides similar results to traditional MS/MS approaches, and has low false positives, especially at the level of sum composition and lipid class.

*Results and Discussion:*

*Comparison of relative concentrations to previous measurements on NIST SRM 1950*

Final normalized values in nmol/mL were compared to both the NIST inter-laboratory study [5] and the LIPID MAPS consortium analysis of NIST SRM 1950 [3]. The values diverged significantly between all three studies for lipids summed at the level of carbons and double bonds (Supplemental Table S4 and Table S5, Supplemental_Comparison.xslx), emphasizing that single point calibration using class representative internal standards in reverse phase is a normalization method and not quantitative. Hence, the advantages of internal standard based normalization are a reduction in variance of measurements and better statistics as discussed in the prior paragraph, but values are not absolute amounts which can be comparable across laboratories and techniques. But other approaches using LMN could be considered semi-quantitative. Because standards and analytes co-elute in separation techniques such as SFC and HILIC, the application of LipidMatch Normalizer along with appropriate correction factors for ionization efficiencies could be semi-quantitative. The median of the absolute relative percent difference (as calculated in Formula 1) for lipids overlapping between this study and the LIPID MAPS study was 138 % with an interquartile range of 274 %. The median percent difference between this study and the NIST inter-laboratory study was 89 %, with an interquartile range of 100 %. The median percent difference between the LIPID MAPS study and NIST inter-laboratory study was 119 % with an interquartile range of 376 %.

The strongest agreement between studies was for this study and the LIPID MAPS study on triglycerides (TGs) (13 % absolute median percent difference, IQR of 25 %), this study and the inter-laboratory study for sphingomyelins (27 % absolute median percent difference, IQR of 87 %), and the LIPID MAPS and inter-laboratory study for ceramides (33 % absolute median percent difference, IQR of 66 %). The most drastic differences between these studies and other studies where when class specific internal standards were not available. For example, when triglycerides were used to normalize cholesterol esters values were over 3-orders of magnitude lower than expected.

*References:*

1. Patterson RE, Kirpich AS, Koelmel JP, Kalavalapalli S, Morse AM, Cusi K, et al. Improved experimental data processing for UHPLC–HRMS/MS lipidomics applied to nonalcoholic fatty liver disease. Metabolomics. 2017;13:142.

2. Koelmel JP, Kroeger NM, Ulmer CZ, Bowden JA, Patterson RE, Cochran JA, et al. LipidMatch: an automated workflow for rule-based lipid identification using untargeted high-resolution tandem mass spectrometry data. BMC Bioinformatics. 2017;18:331.

3. Quehenberger O, Armando AM, Brown AH, Milne SB, Myers DS, Merrill AH, et al. Lipidomics reveals a remarkable diversity of lipids in human plasma,. J Lipid Res. 2010;51:3299–305.

4. Fahy E, Sud M, Cotter D, Subramaniam S. LIPID MAPS online tools for lipid research. Nucleic Acids Res. 2007;35 Web Server issue:W606–12.

5. Bowden JA, Heckert A, Ulmer CZ, Jones CM, Koelmel JP, Abdullah L, et al. Harmonizing lipidomics: NIST interlaboratory comparison exercise for lipidomics using SRM 1950-Metabolites in Frozen Human Plasma. J Lipid Res. 2017;58:2275–88.

*Supplemental Figures:*

a)
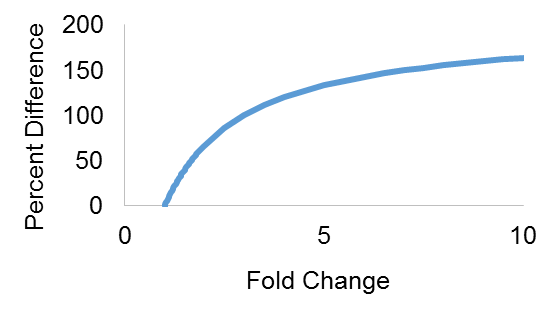


b)
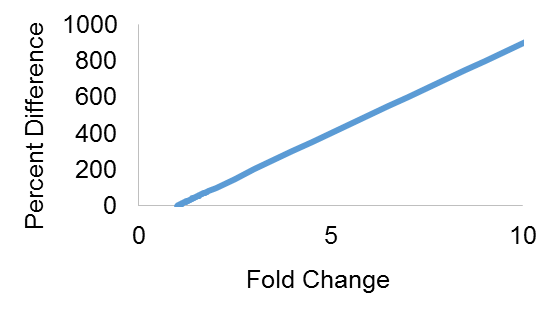


Figure S1a: Fold change (greater than 1) versus percent difference calculated using the average in the formula:

*R*$elative percent difference=\frac{x-y}{\left. Avg(x,y \right)}\times100$

Figure S1b: Fold change (greater than 1) versus percent difference calculated using the minimum in the formula:

*R*$elative percent difference=\frac{x-y}{\left. min(x,y \right)}\times100$


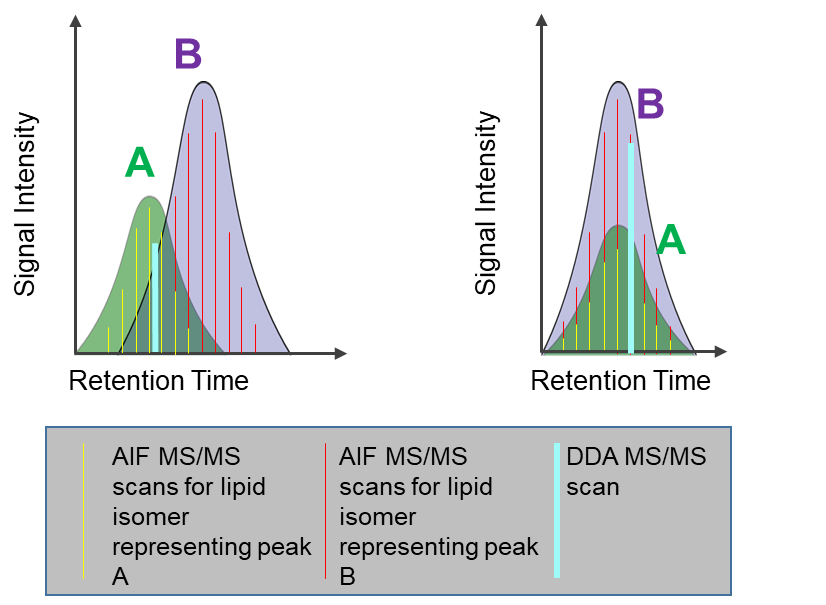


Figure S2: Depiction of MS/MS scans using AIF (red and yellow) and using DDA (white) for two lipid isomers (A and B). An advantage of AIF over data-dependent MS/MS for annotation is that via deconvolution, the correct peak can be assigned to the correct lipid (Figure S1a). In DDA in Figure S1a, if only one MS/MS scan is obtained, there is not enough information to correctly assign which lipid isomer belongs to which peak. If both isomers completely overlap, AIF cannot be used to distinguish the isomers.


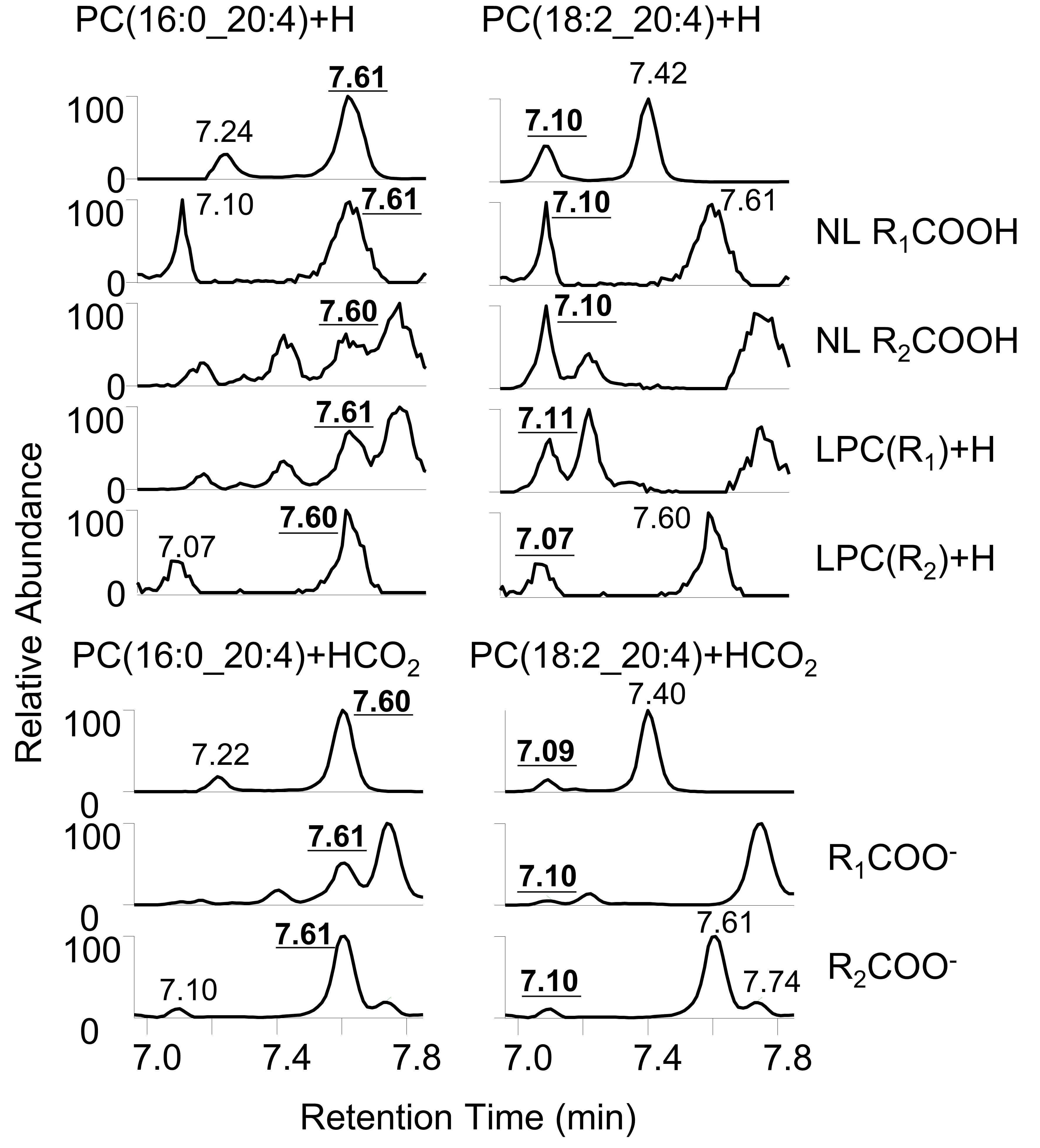


Figure S3: Examples of extracted mass chromatograms for the precursors and fragments of PC(16:0_20:4) and PC(18:2_20:4) as protonated and formate adducts in positive and negative mode, respectively. Fragmentation was obtained by AIF and shows correlation of the elution profile of precursor and fragments for both species. The bold retention time values represent the precursor or precursor fragments, while non-bold represent fragments from the precursor in the adjacent panel.


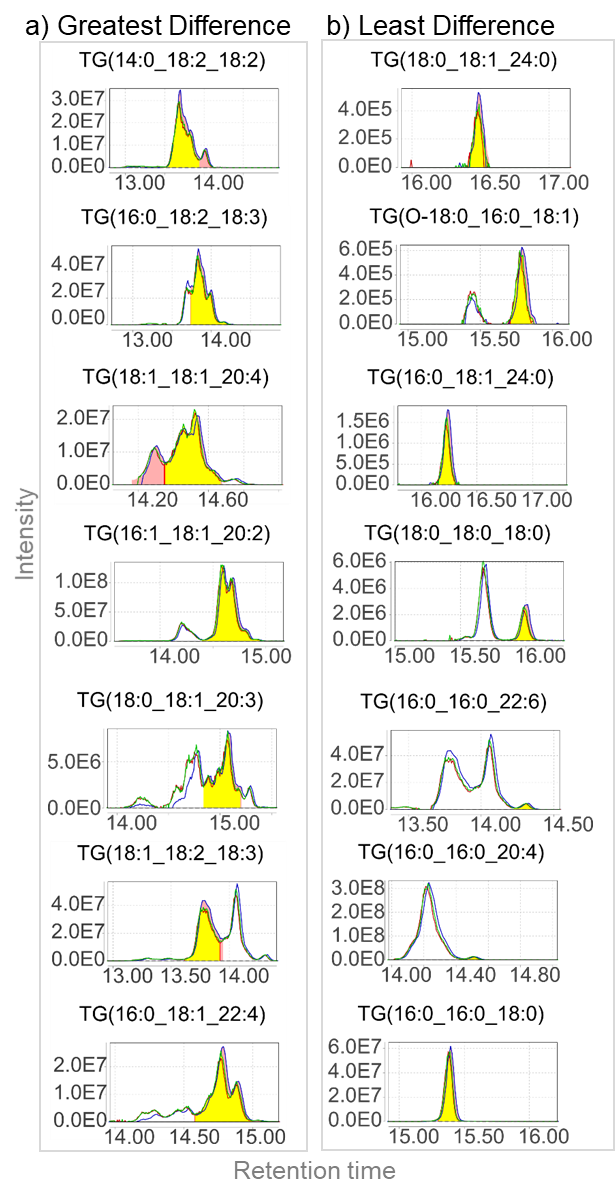


Figure S4: Extracted ion chromatograms (EICs) and peak integration by MZmine of the triglycerides (TGs) with the most (Figure 4a) and least (Figure 4b) percent difference when comparing quantitation using peak height versus peak area.


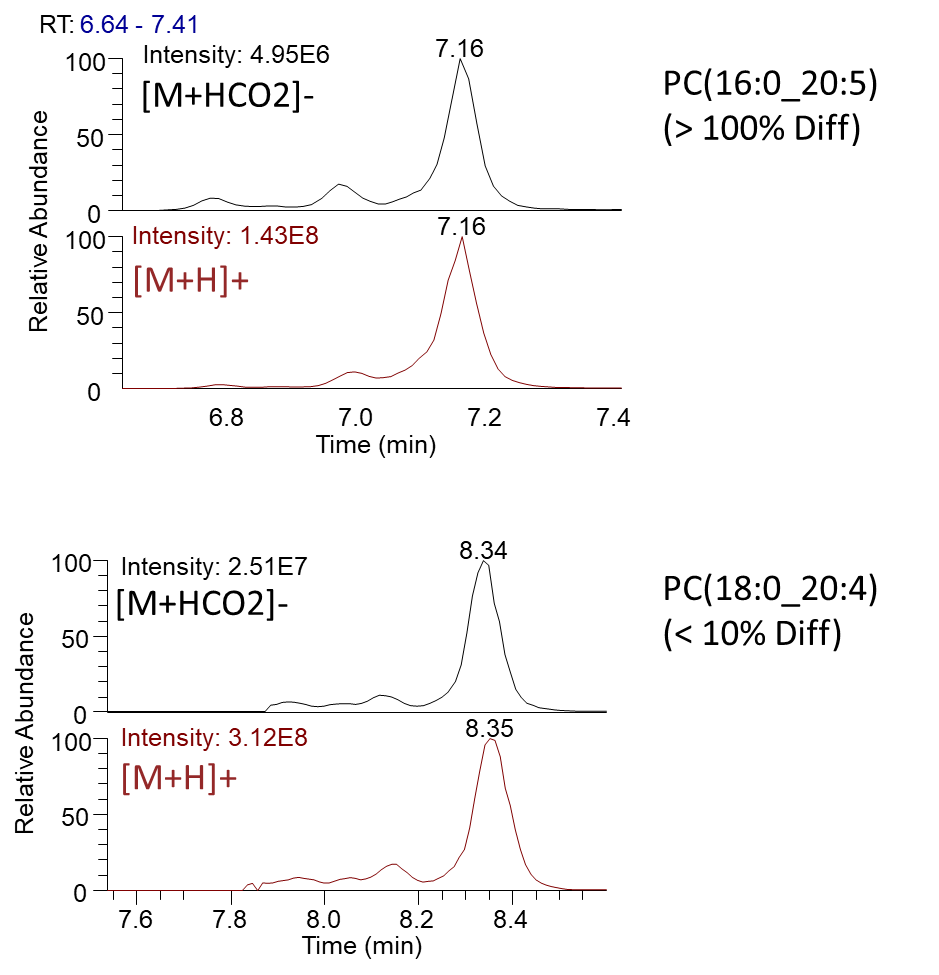


Figure S5: Extracted ion chromatograms (EICs) of PC(16:0_20:5) and PC(18:0_20:4) (the predominant peak at 7.16 and 8.34, respectively). Peaks had similar looking EICs in negative and positive mode, but very different percent differences in concentration between the two polarities, although the same molecular lipid was used for quantification.

*Supplemental Tables:*

Table S-1: Gradient for reverse phase liquid chromatography of lipids. Mobile phase C consisted of 60:40 acetonitrile:water and mobile phase D consisted of 90:8:2 isopropanol:acetonitrile:water, with both containing 0.1% formic acid 10 mM ammonium formate. The flow rate was 500 µL/min.

Table S-2: Mass spectrum scan parameters. Acronyms are automatic gain control (AGC), all-ion fragmentation (AIF), normalized collision energy (NCE), and targeted tandem mass spectrometry (Targeted MS2).

Table S3: The relative percent difference between quantification using different methods or ions

Please see the table of acronyms for lipids.

*The number of species identified by both methods or ions

**The average ± standard deviation of the absolute relative percent difference

***The median relative percent difference (see formula * for calculation of difference)

****The following ratios were used: smooth/not, height/area, pos/neg, and major/[M+Na]^+^

Table S3: The relative percent difference between quantification using different methods or ions (continued)

Please see the table of acronyms for lipids

*The number of species identified by both methods or ions (for example there will be a peak

height and area for all lipids, but only certain lipids were identified in both polarities)

**The average ± standard deviation of the absolute relative percent difference

***The median relative percent difference (see formula * for calculation of difference)

****The following ratios were used: smooth/not, height/area, pos/neg, and major/[M+Na]+
