# Supplementary material for: Software tool for internal standard based normalization of lipids, and effect of data-processing strategies on resulting values
Source: BMC Bioinformatics. 2019 Apr 29;20:217. doi: 10.1186/s12859-019-2803-8 (PMC6489209; doi:10.1186/s12859-019-2803-8)
Supplement: Supplementary file 3 — LipidMatch Normalizer Software. The LMN_Software.zip file contains batch files for lipidomics with MZmine processing and the LipidMatch Normalizer R script. The .zip file also contains files to guide the user in using LipidMatch, which include: A manual and troubleshooting document, and example input and output data (the data used in this paper). For the most up to date version of LipidMatch Normalizer please visit: http://secim.ufl.edu/secim-tools/. (ZIP 126 kb) [file 12859_2019_2803_MOESM3_ESM.zip › LMN_Software/2018_07_19_LMN_Instructions.docx]

**LipidMatch Quant Instructions**

Jason Cochran ([j.a.cochran.cs@gmail.com](mailto:j.a.cochran.cs@gmail.com))

Jeremy Koelmel ([jeremykoelmel@gmail.com](mailto:jeremykoelmel@gmail.com))

Access all tools at:

<http://secim.ufl.edu/secim-tools/>

Or

<https://github.com/JasonCochran/LipidMatchQuant>

View video tutorials at:

<https://www.youtube.com/playlist?list=PLZtU6nmcTb5mQWKYLJmULsfqNy9eCwy7K>

[LipidMatch Tutorial 10: LipidMatch Normalizer](https://www.youtube.com/watch?v=Br7GW5O4LoI&index=12&list=PLZtU6nmcTb5mQWKYLJmULsfqNy9eCwy7K&t=0s)

**Workflow Instructions:**

1. Download R (built and tested using R version 3.3.3): <https://cran.r-project.org/bin/windows/base/old/3.3.3/>
2. Download R-studio (optional): <https://www.rstudio.com/products/rstudio/download/>
3. After downloading the LipidMatch Normalizer zip file, unzip the ‘LipidMatch Normalizer’ folder and place it in a convenient location.
4. Place the input files in a single folder, which can contain other files. See the section titled "Formatting" for formatting information of the input files. A typical quantification data set could include:
   1. Positive Feature Table (only one feature table is required; see Table 1 below)
   2. Negative Feature Table (only one feature table is required; see Table 1 below)
   3. Internal Standards Table (necessary; see Table 2 below)
5. Launch the script titled LMN_v*.R, where * is the version number, using RStudio. The code is contained in the LMN zip file. The code does not need to be modified.
6. To run the script, select all of the code in the browser window (Ctrl A) and click run. The first time running the script, it may take some time for all the packages to be installed by the software; this only occurs during the first run.
7. A pop-up box will appear, select the input file directory made in step 2.
8. The GUI for the LipidMatch Normalizer software will then appear on the screen. Use the buttons to select the Feature Table and Internal Standards Table for quantification. Negative and positive mode data must be run separately (A screen shot of the dialogue box for inputs is shown below (Figure 1)).
9. Utilize the input boxes to provide the following (note that an example is given below of a formatted feature table (see Table 1 below) and internal standards table (see Table 2 below), and the respective values for them):
   1. *m/z* Tolerance: *m/z* tolerance (Da) for matching retention times provided in the internal standards table to retention times (RT) in the feature table. A value of 0.005 would indicate a 0.01 Da window for matching.
   2. RT Tolerance: tolerance for matching retention times provided in the internal standards table to retention times in the feature table.
   3. Sample Start Column: The first peak area/height column in the feature table.
   4. Sample End Column: The last peak area/height column in the feature table. Note that all sample columns, with their respective peak heights or areas, should be adjacent without any other columns between.
   5. *m/z* Column: The column in the feature table with *m/z* values for all features.
   6. RT Column: The column in the feature table with retention times for all features.
   7. Class ID Column: The column in the feature table with class information for each feature (e.g., PC, LPC, PE, LPE, TG, etc.). Note that the acronym or name for each lipid class should match exactly the respective acronym or name of each lipid class in the internal standards table. Not all lipid classes in the feature table need to be represented in the internal standard table, and vice versa (although this is ideal).
   8. Adduct ID Column: The column in the feature table with adduct information for each feature (e.g., [M+H]^+^, [M+NH_4_]^+^, [M-H]^-^, etc.). Note that the acronym or name for each lipid adduct should match exactly the respective acronym or name of each lipid adduct in the internal standards table. Not all adducts in the feature table need to be represented in the internal standard table, and vice versa (although this is ideal).
   9. Numeric Data Start Row: The row in which numeric data starts for the following input columns above (denoted by the letters c, d, e and f above): peak areas/heights, *m/z* values and retention time values).
   10. Sample Grouping Row: The row with variable or grouping names for each sample, sample groupings are not necessary in which case put an arbitrary row.

Figure 1: Input Dialogue Box


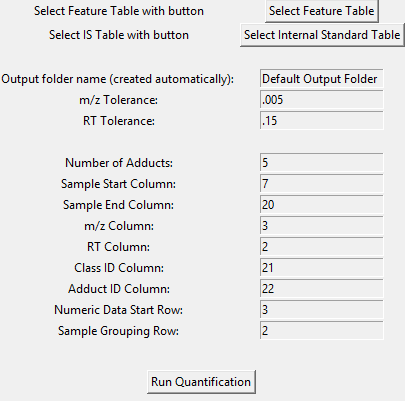


1. When all of the necessary information is inputted, hit the “Run Quantification” button.
2. When the software is finished running, an output folder is generated in the input folder directory. A quantification table and a table with the internal standards found in the feature table, both as .csv files, should be generated in this output folder.
   1. If nothing was displayed in the output, check for errors in the R console. (They appear as text in the console. R errors will appear red, but not have the message containing "Warning" (those are not errors). Check the troubleshooting table for additional assistance.

**Formatting of Feature Table:**

The formatted feature table below demonstrates user parameters in the LMN dialogue window. Any number of rows can be included before the numeric data starts (with at least 2 rows). Hence, the data must start in at least row 3. In the example provided, the data starts in row 3 (Numeric Data Start Row). Note that in this case, an arbitrary row filled with NAs was inserted to make sure that there were at least 2 rows before the numeric data started. Any number of columns can be added before and after the samples. In the example provided, the sample peak areas start in column 4 (Sample Start Column) and end in column 6 (Sample End Column). The "*m/z* Column" is 2, the "RT Column" is 3, the Class ID Column is 8 and the Adduct ID Column is 9.

Table 1: Feature Table

| row ID | *m/z* | RT | Sample1 | Sample2 | Sample3 | ID_Ranked | Class | Adduct | … |
| --- | --- | --- | --- | --- | --- | --- | --- | --- | --- |
| NA | NA | NA | NA | NA | NA | NA | NA | NA | … |
| 980 | 662.5720 | 9.0 | 1.3E+06 | 7.7E+05 | 1.1E+06 | 1_DG(18:1_20:3)+NH4 | DG | [M+NH_4_]^+^ | … |
| 19 | 812.6160 | 8.7 | 1E+09 | 1E+09 | 1E+09 | 2_PC(18:0_20:3)+H \| 1_PC(18:0_20:3)+H | PC | [M+H]^+^ | … |
| … | … | … | … | … | … | … | … | … | … |

**Formatting of Internal Standards Table:**

An example formatted internal standards table is shown below. Unlike the feature table, the format for the internal standard table is not flexible. The first column in the internal standard table is the internal standards (IS) names which should begin in the second row. The value in the first row can be anything but blank. The internal standard names are only used to display the internal standards used to quantify each feature, and therefore, there are no rules for naming conventions.

The retention time (RT), is the exact retention time for each IS, which should be consistent across all samples. This can be the median RT, average RT, or a representative RT across all samples. The *m/z* values reported should be for each adduct and can be calculated using the internal standard name with *LipidPioneer* (<http://secim.ufl.edu/secim-tools/lipidpioneer/>). If no peak is observed for a certain *m/z* value, an NA can be used instead. In the example shown below, the "Number of Adducts" in the user input dialogue box would be 3. The user can add or remove as many adducts as deemed necessary, with each adduct inserted in an additional adjacent column.

For the classes column, which should be the column following the adduct columns, lipid classes which are to be quantified using each internal standard should be entered. Each lipid class to be quantified using the class representative IS should be separated by a space. The class naming styles should be exactly the same as in the feature table class column. After the "Classes" column, the samples should be included in the same order as the samples in the feature table, with the same number of samples. For each row, the amount of IS for the respective sample should be included. Note that this amount can be in any units of concentration (e.g., µg/g tissue, nmol/mL, etc.), and the resulting concentrations in the final output will be in the same units.

Note that the names of the columns are irrelevant, and that any blank cells should be filled with "NA" as below.

Table 1: Internal Standards Table

| Name | RT | *m/z* (Pos) | *m/z* (Pos) | *m/z* (Pos) |  | Classes | Sample1 | Sample2 | Sample3 |  |
| --- | --- | --- | --- | --- | --- | --- | --- | --- | --- | --- |
| NA | NA | [M+H]^+^ | [M+NH_4_]^+^ | [M+Na]^+^ |  | NA | NA | NA | NA | … |
| PE(17:0/17:0) | 9.38 | 720.5538 | NA | 742.5357 |  | PE ether-PE | 24.25 | 24.25 | 24.25 | … |
| PG(17:0/17:0) | 8.63 | 751.5483 | 768.5749 | 773.5303 |  | PG | 23.25 | 23.25 | 23.25 | … |
| PC(17:0/17:0) | 9.22 | 762.6007 | NA | 784.5826 |  | PC ether-PC | 23 | 23 | 23 | … |
| LPC(17:0) | 2.16 | 510.3554 | NA | 532.3373 |  | LPC ether-LPC | 35 | 35 | 35 | … |
| PS(17:0/17:0) | 8.70 | 764.5436 | NA | NA |  | PS | 23 | 23 | 23 | … |
| SM(d18:1/17:0) | 8.02 | 717.5905 | NA | 739.5724 |  | SM | 24.5 | 24.5 | 24.5 | … |
| Cer(d18:1/17:0) | 8.68 | 552.5350 | NA | 574.5169 |  | Cer | 32.5 | 32.5 | 32.5 | … |
| TG(15:0/15:0/15:0) | 14.38 | NA | 782.7232 | 787.6786 |  | TG ether-TG | 6.5 | 6.5 | 6.5 | … |
| 13C2-Cholesterol | 7.11 | NA | 371.3584 | NA |  | CE | 45 | 45 | 45 | … |
| … | … | … | … | … |  | … | … | … | … |  |

**FAQ:**

Q: What can I use this software for?

*A: The software can be used in liquid chromatography mass spectrometry based lipidomic studies where it is desirable to use class representative internal standards for normalization of your lipid signals.*

Q: Will feature [x] be included in the software? I think it would be a really good idea.

*A: Contact the developers through the Github page for ideas and questions! We are more than happy to hear your thoughts.*

Q: I found a bug (or am I doing something wrong?)
*A: Report all bugs on our Github page. Please include the error message received, R workspace, the feature table, the internal standards table, a screen shot of the input GUI with user parameters (as in Figure 1), and a clear explanation of the issue encountered. You can also email Jeremy Koelmel (jeremykoelmel@gmail.com) or Jason Cochran (j.a.cochran.cs@gmail.com).*

**Troubleshooting:**

Please make sure you are using the latest version of the LipidMatch Normalizer script (http://secim.ufl.edu/secim-tools/).

| **Issue** | **Solution** |
| --- | --- |
| The software outputs the ‘standardsFound.csv’ file correctly but not the quantified amounts file. | There is most likely an error in the class/adduct identification columns. Ensure that the column indices are correctly indicated in the LMN GUI. If this is correct, please ensure that formatting of the tables is correct. |
| The software doesn’t do anything! | Ensure you have the internal standards table and the feature table correctly loaded. Also check that the column and row numbers are correctly entered. |
