# Supplementary figures and images for: Software tool for internal standard based normalization of lipids, and effect of data-processing strategies on resulting values
Source: BMC Bioinformatics. 2019 Apr 29;20:217. doi: 10.1186/s12859-019-2803-8 (PMC6489209; doi:10.1186/s12859-019-2803-8)

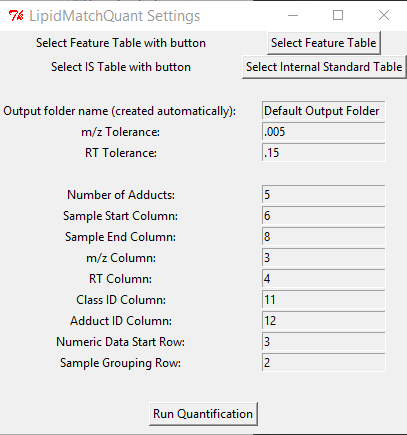

Supplement: Supplementary file 3 — LipidMatch Normalizer Software. The LMN_Software.zip file contains batch files for lipidomics with MZmine processing and the LipidMatch Normalizer R script. The .zip file also contains files to guide the user in using LipidMatch, which include: A manual and troubleshooting document, and example input and output data (the data used in this paper). For the most up to date version of LipidMatch Normalizer please visit: http://secim.ufl.edu/secim-tools/. (ZIP 126 kb) [file 12859_2019_2803_MOESM3_ESM.zip › LMN_Software/Example_Files/NIST_SRM_1950/SRM1950_Example_Parameters.PNG]
